# Prevention of vitamin D deficiency in children following cardiac surgery: study protocol for a randomized controlled trial

**DOI:** 10.1186/s13063-015-0922-8

**Published:** 2015-09-09

**Authors:** J. Dayre McNally, Katie O’Hearn, Margaret L. Lawson, Gyaandeo Maharajh, Pavel Geier, Hope Weiler, Stephanie Redpath, Lauralyn McIntyre, Dean Fergusson, Kusum Menon, on behalf of the Canadian Critical Care Trials Groups

**Affiliations:** Department of Pediatrics, Faculty of Medicine, University of Ottawa, Children’s Hospital of Eastern Ontario, Ottawa, Canada; Research Institute, Children’s Hospital of Eastern Ontario, 401 Smyth Road, Ottawa, ON K1H 8L1 Canada; Division of Cardiovascular Surgery, University of Ottawa, Ottawa, Canada; School of Dietetics and Human Nutrition, McGill University, Montreal, QC Canada; Department of Medicine (Division of Critical Care), Ottawa Hospital Research Institute (OHRI), University of Ottawa, Ottawa, ON Canada; Department of Epidemiology and Community Medicine, Ottawa Hospital Research Institute (OHRI), University of Ottawa, Ottawa, ON Canada

**Keywords:** vitamin D, pediatrics, congenital heart disease, randomized controlled trial

## Abstract

**Background:**

Vitamin D is a pleiotropic hormone important for the recovery of organ systems after critical illness. Recent observational studies have suggested that three out of every four children are vitamin D deficient following cardiac surgery, with inadequate preoperative intake and surgical losses playing important contributory roles. Observed associations between postoperative levels, cardiovascular dysfunction and clinical course suggest that perioperative optimization of vitamin D status could improve outcome. With this two-arm, parallel, double blind, randomized controlled trial (RCT), we aim to compare immediate postoperative vitamin D status in children requiring cardiopulmonary bypass for congenital heart disease who receive preoperative daily high dose vitamin D supplementation (high-dose arm) with those who receive usual intake (low-dose arm).

**Methods/Design:**

Eligibility requirements include age (>36 weeks, <18 years) and a congenital heart defect requiring cardiopulmonary bypass surgical correction. Enrollment of 62 participants will take place at a single Canadian tertiary care center over a period of 2 years. Children randomized to the high-dose group will receive age-based dosing that was informed by the Institute of Medicine (IOM) daily tolerable upper intake level (<1 year old = 1,600 IU/day, >1 year old = 2,400 IU/day). Children in the low-dose arm will receive usual care based on IOM recommendations (<1 year old = 400 IU, >1 year old = 600 IU). The primary outcome measure is immediate postoperative vitamin D status, using blood 25(OH)D.

**Discussion:**

Maintaining adequate postoperative vitamin D levels following surgery could represent an effective therapy to speed recovery following CHD surgery. The proposed research project will determine whether preoperative supplementation with a dosing regimen based on the IOM recommended daily upper tolerable intake will prevent postoperative vitamin-D deficiency in the majority of children. The results will then be used to inform the design of a large international RCT exploring whether preoperative optimization of vitamin D status might improve short and long-term outcomes in this vulnerable population.

**Trial Registration:**

Clinicaltrials.gov Identifier - NCT01838447

Date of registration: 11 April 2013

**Electronic supplementary material:**

The online version of this article (doi:10.1186/s13063-015-0922-8) contains supplementary material, which is available to authorized users.

## Background

Congenital heart disease (CHD) is a common condition with an estimated prevalence of 1 per 100 in the general population. A significant proportion of these pediatric patients require one or more corrective surgeries over their lifetime, collectively leading to 15,000 procedures per year in North America [1]. Postoperatively, these patients suffer significant morbidities, which may include a pronounced systemic inflammatory response, multiple organ failure, electrolyte disturbances, arrhythmia, infection and endocrine imbalances [2–5]. Interventions that prevent or modulate postoperative pathophysiology may prevent illness, speed recovery, and decrease chronic morbidity in this high-risk pediatric population.

Vitamin D status is well recognized as important to calcium homeostasis and musculoskeletal health. The importance of Vitamin D and its physiological effects are well understood in the context of hypocalcemia [6, 7]. Briefly, as serum calcium falls, the parathyroid increases parathyroid hormone (PTH) secretion leading to activation of vitamin-D through an inducible renal enzyme. The inducible renal enzyme works to convert serum 25-hydroxyvitamin D [25(OH)D] to 1,25 -dihydroxyvitamin D [1,25(OH)_2_D] and this active metabolite circulates to the bone, gut and kidneys to restore homeostasis. It is now known that many cell types do not rely entirely on kidney production and have enzymes capable of converting 25(OH)D to its active form for both autocrine or paracrine use. Circulating 25(OH)D is well accepted as the best marker for evaluating vitamin D status in the majority of health settings, including the general ICU population [8, 9]. The generally accepted thresholds for defining vitamin D sufficiency is 75 nmol/L, with deficiency defined as below 50 nmol/L, and severe deficiency at 25 to 30 nmol/L).

Increasingly, vitamin D is accepted as a pleiotropic hormone important for the functioning of organ systems central to critical illness pathophysiology, including electrolyte homeostasis, cardiovascular health, inflammation and innate immunity [10–12]. This has lead to the hypothesis that its deficiency might represent a modifiable risk factor for critical illness. A growing number of observational studies in adult cardiovascular and intensive care populations have investigated this hypothesis, and these studies have reported high vitamin D deficiency rates and associations between hormone level and organ dysfunction, health resource utilization and mortality [11, 13–20]. Further, a single moderately sized, interventional study on critically ill adults (VITdAL-ICU) suggests that rapid repletion of vitamin D may improve outcomes [21]. This trial randomized 475 critically ill adults to an initial enteral 540 000 IU cholecalciferol loading dose (followed by monthly 90,000 IU) or placebo doses. In this study there was a nonsignificant absolute risk reduction in hospital mortality of in the vitamin D arm (7.0 %, *P* = 0.10). However, in the predefined subgroup of patients with vitamin D levels below 30 nmol/L at baseline this absolute difference became larger and statistically significant (−17.5 %, *P* = 0.01). Although, large pediatric observational studies have also documented high rates of vitamin D deficiency and associations between hormone levels and clinical course within the PICU [22, 23], a high dose interventional vitamin D trial in the PICU has yet to be undertaken [24].

Patients with CHD requiring surgery have been investigated as subgroups within large PICU studies and as distinct populations [22, 25]. Analysis of the CHD patients enrolled in a large multicenter Canadian PICU reported a 70 % deficiency rate and associations between vitamin D level and clinical course [22]. Further, Graham *et al*. confirmed these observations with a secondary analysis of postoperative blood specimens, reporting not only that 84 % of neonates with CHD were vitamin D deficient postoperatively, but that lower levels were associated with inotropic requirements [25]. In addition, in a prospective longitudinal study, we calculated an 85 % vitamin D deficiency rate in the CHD population immediately following surgery, as well as an association between deficiency and postoperative fluid and catecholamine requirements [26]. Mechanistic studies determined the high rate of vitamin D deficiency to be secondary to borderline normal preoperative 25 hydroxyvitamin D (25OHD) levels and an acute 40 % intraoperative decline due to cardiopulmonary bypass (CPB), consistent with that described in an adult CPB study [27]. In summary, the available data suggest that most CHD patients are vitamin D deficient following cardiac surgery and that the immediate postoperative levels are associated with subsequent clinical course.

A role for vitamin D in critical illness has biological plausibility, as there are multiple mechanisms through which deficiency could cause secondary pathophysiology. Hypocalcemia is a common problem following CHD repair (30 %) and calcium replacement is associated with morbidity and mortality [5]. Adult and pediatric ICU studies have shown that critically ill patients with hypocalcemia are more likely to have abnormalities of their vitamin D axis, including low 25OHD, hypoparathyroidism and/or renal dysfunction [28–30]. A role for vitamin D in cardiac health can be found in case reports and case series describing cardiomyopathy secondary to isolated severe vitamin D deficiency [6, 31–33]. A recent RCT of vitamin D supplementation in outpatient pediatric congestive heart failure showed improved cardiac function with a higher daily dose of vitamin D [34]. Additionally, cardiac surgery with cardiopulmonary bypass uniformly leads to a postoperative systemic inflammatory response syndrome [35, 36]. There is good evidence that vitamin D metabolites play important immunomodulatory roles mediated through functional vitamin D receptors present on all major immune cell types [37–39]. Vitamin D signaling is also known to play a role in innate immunity, such as in the production of cathelicidins [40–42]. Cathelicidins, important endogenous antimicrobial peptides, provide protection against multiple viral and bacterial pathogens. Prevention of vitamin D deficiency could decrease nosocomial infections among CHD patients through improved innate immunity.

The current body of knowledge suggests that optimization of vitamin D status prior to and following CHD repair could improve clinical outcomes through reduced inflammation, fewer nosocomial infections, improved cardiac function, and faster postoperative rehabilitation and physical functioning [11, 13–17, 43–45]. However, before these findings can be translated into clinical practice, a number of unknowns must be addressed. For instance, there have been no interventional studies establishing that prevention of postoperative vitamin D deficiency improves clinical outcomes in CHD patients. In addition, attempts to perform a large RCT would be premature because a dosing regimen that prevents postoperative vitamin D deficiency has not yet been identified. Moreover, there have been no vitamin D dosing studies or guidelines developed specific to the CHD population; presently children with CHD receive the same advice regarding supplementation as healthy children [46]. Although it is tempting to extrapolate recent safety data from high dose vitamin D studies on healthy children to the CHD population, this may be inappropriate. CHD patients have unique metabolic demands, organ dysfunctions, as well as known and unknown genetic abnormalities that potentially make them more or less susceptible to vitamin D [47–50]. To begin addressing these knowledge gaps, we have designed a pilot dose evaluation randomized controlled trail (RCT) with the goal of identifying a supplementation regimen that safely prevents postoperative vitamin D deficiency in children requiring cardiopulmonary bypass for CHD.

### Objectives and hypotheses

#### Hypothesis

In pediatric patients requiring surgery for CHD, preoperative supplementation with a daily high dose vitamin D regimen, modelled on the Institute of Medicine (IOM) Tolerable Upper Intake Level (UL) [46], will significantly reduce postoperative vitamin D deficiency, when compared with usual care.

#### Study objectives

The primary and secondary objectives are as follows:

1. The primary objective is to perform a double-blind RCT to determine whether the preoperative administration of a daily high dose of vitamin D, based on the UL from IOM [46], compared with usual care, results in a significant reduction in postoperative vitamin D deficiency in a pediatric population with CHD.
2. Secondary objectives
  - Determine whether the preoperative regimen of daily high dose vitamin D, compared with usual care, results in a greater number of vitamin D related adverse events (hypercalcemia, hypercalciuria).
  - Determine whether the preoperative regimen of daily high dose vitamin D, compared with usual care, improves established markers of vitamin D axis functioning (active hormone levels, cardiac function).
  - Determine the barriers and feasibility of conducting a larger phase III RCT evaluating whether vitamin D supplementation improves clinical outcomes in children who require CHD surgery (blinding, recruitment, and compliance).

## Methods/Design

The Heart and Stroke Foundation of Canada is the Sponsor for the trial. The study will be conducted in accordance with the ethical principles guided by Tri-Council Policy and the Declaration of Helsinki. The protocol is approved by Health Canada (no objection letter, Protocol #VitaminDinCHD-01) and the Children’s Hospital of Eastern Ontario Research Ethics Board (REB reference: 13/03E). The trial will comply with the principles of Good Clinical Practice and will be carried out in accordance with applicable legislation and the Standard Operating Procedures of the CHEO Research Institute. The trial will be reported in line with the Consolidated Standards of Reporting Trials (CONSORT) 2010 guidelines [51] and the Standard Protocol Items: Recommendations for Interventional Trials (SPIRIT) checklist [52] (Additional file 1). Protocol amendments will be communicated as necessary to those involved with the study. A structured summary of the trial is provided in Table 1.

**Table 1:** World Health Organization Trial Registration Data Set – Structured Summary

Data category	Information
Primary registry, trial identifying #	Clinicaltrials.gov Identifier - NCT01838447
Date of registration in primary registry	April 11, 2013
Secondary indentifying numbers	HICCUPS, VitaminDinCHD-01, 13/03E
Sources of monetary support	Heart and Stroke Foundation of Canada Operating Grant, Children’s Hospital of Eastern Ontario Research Institute
Primary sponsor	Heart and Stroke Foundation of Canada
Secondary sponsor	Children’s Hospital of Eastern Ontario Research Institute
Contact for public queries	JDM, Pediatric Critical Care, Children’s Hospital of Eastern Ontario, Ottawa, Canada
Contact for scientific queries	JDM, Pediatric Critical Care, Children’s Hospital of Eastern Ontario, Ottawa, Canada
Public title	Prevention of vitamin D deficiency after congenital heart disease surgery: a dose evaluation trial
Scientific title	Prevention of vitamin D deficiency in children following cardiac surgery: a study protocol for a randomized dose evaluation trial
Country of recruitment	Canada, single academic center
Health problem under investigation	Prevention of vitamin D deficiency after congenital heart disease surgery
Key inclusion and exclusion criteria	Ages eligible for study: Corrected gestational age >36 weeks and <18 years.
	Inclusion criteria: has CHD requiring surgical correction with cardiopulmonary bypass within 12 months
	Exclusion criteria: born at <32 weeks gestational age; cardiac or gastrointestinal disease preventing enteral feeds or drug administration prior to surgery; has confirmed or suspected Williams syndrome; proposed surgery to take place at another center
Study type	Single center, double blind, parallel, randomized, controlled dose evaluation trial
Date of first enrollment	June 2013
Target sample size	62
Recruitment status	Recruiting as of June 2015
Primary outcome	Using immediate postoperative vitamin D status, (that is blood 25OHD) we will determine whether the preoperative administration vitamin D compared with usual care results in a significant reduction in postoperative vitamin D deficiency in a pediatric population with CHD.
Key secondary outcomes	Vitamin D related adverse events (that is hypercalcemia, hypercalciuria); vitamin D axis functioning (active hormone, cardiac function); study feasibility

### Study design

The trial is a single center, double blind, parallel, randomized, controlled dose evaluation trial comparing the efficacy and safety of two vitamin D dosing regimens in the prevention of postoperative vitamin D deficiency in children undergoing surgery for CHD.

### Study population

#### Inclusion

Inclusion criteria are as follows:

1. Between 36 weeks gestational age and 18 years
2. CHD requiring surgery within the next 12 months
3. CHD requiring surgical correction with cardiopulmonary bypass (CBP)

#### Exclusion

Exclusion criteria are as follows:

1. Born at less than 32 weeks gestational age
2. Cardiac or gastrointestinal disease prevented enteral feeds or drug administration prior to surgery
3. Confirmed or suspected William’s syndrome a neurodevelopmental genetic disorder with symptoms that include cardiovascular problems and high blood calcium
4. Proposed surgery to take place at another center (outside of CHEO)

#### Justification of eligibility criteria

In our previous study, CHD patients who did not receive CPB had a minimal (<10 %) intraoperative drop in 25OHD. Although prevention of vitamin D deficiency is important for these patients, they do not require preoperative elevation into the upper normal physiological range. Very premature infants (<32 weeks) are at significantly increased risk for nephrocalcinosis [53, 54]. CHD patients with Williams syndrome have a genetic susceptibility to hypercalcemia and current guidelines recommend against any vitamin D supplementation [49, 55].

### Study drug

#### Study drug distribution

Europharm will provide the study drug (vitamin D) in the required concentrations, prepared in indistinguishable vials for blinding purposes. The study drug will be analyzed as per Health Canada regulations. The pharmacy will administer the study drug according to randomization, participant age, and whether the patient is being breast-fed or formula fed. Infants with CHD assigned to the usual care arm will be given either a placebo (0 IU/mL) solution if they are receiving vitamin D as part of formula, or they will be given a 400 IU/mL solution if they are breast-fed.

### Proposed supplement doses (interventions) to be tested and rationale

#### Treatment groups

The doses for evaluation have been modelled on the two age-specific intake levels recommended by the IOM [46] (Table 2).

**Table 2:** Vitamin D supplementation strategy

A: Breastfed infant or over 12 months of age					
Age Group	Volume	Standard Dose Group		High Dose Group	
	mL	IU per day	Vial Concentration	IU per day	Vial Concentration
0 to 1 year	1	400 IU	400 IU/mL	1600 IU	1600 IU/mL
1 to 17 years	1	600 IU	600 IU/mL	2400 IU	2400 IU/mL
B: Formula fed and under 12 months of age					
Age Group	Volume	Standard Dose Group		High Dose Group	
	mL	IU per day	Vial Concentration	IU per day	Vial Concentration
0 to 1 year	1	None^a^	Placebo, 0 IU/mL	1200 IU^a^	1200 IU/mL

^a^Does not include the vitamin D intake from formula (400 IU/day)

^b^We will not increase vitamin D levels in those children who turn 1 year old after initiating the study drug

Further details on the study drug: (1) Isoform - cholecalciferol (D3), (2) Route – Enteral, (3) Form – Solution (4) Frequency – Daily, (5) Duration – from time of CHD diagnosis to day of operation (started no more than 6 months prior to surgery date). Euro-pharm has agreed to provide the study drug in 50-mL vials with the following concentrations to achieve blinding: placebo, 400 IU/mL, 600 IU/mL, 1200 IU/mL, 1600 IU/mL, and 2400 IU/mL.

The high-dose group is based on the age-specific UL. These doses were chosen to elevate 25OHD well above 50 nmol/L, while minimizing risk of vitamin D toxicity (for example, hypercalcemia or hypercalciuria). Patients under 1 year of age will receive 1,600 IU/day, whereas those over 1 year of age will receive 2,400 IU/day. Infants under 6 months of age in the high-dose group will receive 600 IU/day more than the UL from IOM, whereas those between 6 and 12 months will receive 100 IU/day more than the UL.

The usual care group will receive Adequate Intake (AI) for infants and Recommended Dietary Allowance (RDA) for children over 1 year old (<1 year old, 400 IU; >1 year old, 600 IU/day). These doses were chosen by IOM to achieve blood 25OHD levels above 50 nmol/L in the vast majority of the healthy population.

### Rationale for inclusion of the usual care arm

Given that children with CHD receive the same vitamin D supplementation advice as healthy children (by default), it is tempting to conclude that usual care dosing will be insufficient to prevent vitamin D deficiency. Unfortunately, this conclusion may be wrong for the following reasons: (i) only 50 % of previous study participants indicated daily vitamin D intake at or above 400 IU [22], (ii) compliance with vitamin D supplementation may be poor without motivation (research studies, vitamin D-related disease), (iii) there is often uncertainty about vitamin D intake by caregivers, and (iv) recommendations for usual care recently increased to 600 IU for children above 1 year old [46]. Given potential safety concerns regarding high doses of vitamin D in diseased population, it would be appropriate to properly evaluate the efficacy of usual care under ideal circumstances.

In addition, it is important to assess the baseline risk for vitamin D-related adverse events in CHD patients receiving the usual (standard of care) dosing. Although studies on healthy children have not identified adverse events (for example, hypercalcemia or hypercalciuria) with doses at and slightly above the IOM tolerable upper intake level [56, 57], diseased populations including those with CHD may be predisposed at lower 25OHD levels.

### Rationale for preoperative daily enteral approach

Our previous prospective study demonstrated that post-CHD surgery, vitamin D deficiency occurs due to borderline normal preoperative values and a consistent CPB-induced intraoperative decline [26]. As there is no intravenous form of cholecalciferol, or 25OHD, maintenance of appropriate postoperative levels will require elevation of preoperative levels into the high normal range using enteral supplementation. Two basic approaches for enteral restoration and maintenance of vitamin D stores have been described. First, representing usual care, is the daily consumption of a relatively low dose of cholecalciferol (400 to 4,000 IU/day). The second option is a single or divided megadose of vitamin D (100,000 to 600,000 IU) given intermittently throughout the year [58, 59]. As safety concerns regarding the megadose approach in children have not been adequately addressed, we have chosen to evaluate regimens based on daily consumption [60].

### Anticipated duration of study drug and perioperative 25OHD levels

#### Duration of study drug

Vitamin D supplementation is generally initiated within a week of birth, so the duration of preoperative therapy would be from birth (or diagnosis of CHD) to the time of surgery. Timing of surgery is very dependent on the type of CHD lesion. Consistent with the literature, our recent observational study of perioperative vitamin D status demonstrated 8 months as the median age at surgery, with 2.4 months being the 25th percentile. Based on these findings, we anticipate that it should be possible for 75 to 80 % of patients to receive study drug for more than 2 months (the time required to achieve a new 25OHD steady state with high dose daily supplementation). Whether this duration of study drug can actually be achieved preoperatively and the 25OHD levels achieved will be reported as part of the pilot study.

#### Perioperative 25OHD levels

Given the 40 % intraoperative decline, preoperative levels above 90 nmol/L will be required to maintain postoperative levels above 50 nmol/L (the value at which sufficient substrate to synthesize the active metabolite is available). The ability of certain vitamin D intake levels to achieve this preoperative value can be inferred from recently completed dosing studies on healthy children level [56, 57]. These studies have shown that usual care dosing for 2 to 3 months will achieve preoperative levels of 90 nmol/L in only 40–50 %. In contrast, studies evaluating doses approximating our higher daily intake level (1600 IU/day) achieved mean 25OHD levels of 130 to 150 nmol/L, suggesting that 80 % of CHD patients could achieve preoperative levels of 90 nmol/L or above.

### Subject recruitment

Potentially eligible study participants will be identified in the ambulatory clinics (cardiology, cardiovascular) or inpatient wards (including pediatric intensive care and neonatal intensive care unit) by a member of the study staff. A research coordinator will provide study information and obtain informed consent as well as applicable assent from each participant (Additional file 2). Study staff will provide support to participants and encourage adherence to intervention protocols.

### Randomization, blinding and stratification procedures

We will use a computer-generated randomization sequence. Only the CHEO pharmacy will have access to the randomization sequence and will be responsible for participant randomization and allocation. Only the pharmacist will know the identity of the study drug administered to specific patients. Given the expected recruitment (two to three per month) and potential impact of season on 25OHD, randomization will be performed in permuted blocks (four within each stratum). We will blind patients, families, investigators, hospital staff, and research personnel to treatment arm. Blinding was considered necessary to avoid (i) families altering the outpatient dose and (ii) a number of secondary outcome measures are that are potentially subjective (echocardiography, timing of extubation, need for fluids or catecholamine infusion). Within each age group, the two interventions will be indistinguishable (vial, volume, color, taste, consistency and smell).

Participants will be stratified into whether or not they are expected to receive at least 8 weeks of study drug prior to surgery. This stratification should guarantee that an equal number of CHD patients who will not receive 8 weeks of oral dosing end up in both the high and low dose arms. We will further stratify by age (under or over 1 year of age).

### Co-interventions

We will not protocolize postoperative co-interventions as the study is single center and CHEO has standardized approaches to the common postoperative complications and adverse events (for example, hypocalcemia, junctional ectopic tachycardia, necrotizing enterocolitis constipation, sedation, catecholamine administration, *etcetera*). As the study is blinded, protocolization of co-interventions is less relevant and differences should relate to random chance or drug effects.

### Diagnostic and clinical outcome measures

#### Blood 25OHD

The primary objective will be evaluated using immediate postoperative blood 25OHD concentrations (collected on the day of ICU admission) with a level lower than 50 nmol/L used to define deficiency [61, 62]. This is a well-established cut-off based on (i) the knowledge that the parathyroid and renal organs need to compensate for 25OHD levels below 50 nmol/L and (ii) clinical studies that have shown increased risk of bone, cardiovascular, immune and other disease entities once concentrations fall below this level. The 25OHD will be determined using a LC-MS/MS assay from a laboratory participating in the Vitamin D External Quality Assessment Scheme (DEQAS) [22, 63].

#### Vitamin D-related adverse events

We will report, by intervention, on the occurrence of clinically significant adverse events. However, a measurable difference in clinically significant adverse events between the high dose and usual care arms of the study is unlikely. Therefore, to enhance our ability to evaluate for potential toxicity, we will use two well-accepted surrogate outcome measures:

1. *Hypercalcemia:* This will be defined as an ionized calcium level above 1.40 mmol/L (or above 1.45 mmol/L for children under 8 weeks) (Table 3). We will evaluate for hypercalcemia in blood collected immediately before surgery and throughout the postoperative course (measurements are a standard of care, and any single episode of hypercalcemia not related to parenteral administration of calcium will be considered an adverse event) [64].

**Table 3:** Safety thresholds for ionized calcium, total corrected calcium and elevated calcium-creatinine ratio

Table 3A: Age specific thresholds for pH corrected ionized calcium levels	
Age (months)	95th % (mmol/L)
<2	1.45
>2	1.4
Table 3B: Threshold for corrected total calcium level	
Age (months)	mmol/L
<3	2.8
>3	2.7
Table 3C: Age specific thresholds for elevated calcium-creatinine ratio	
Age (year)	95th % Ca/Cr ratio (mmol/mmol)
<1	2.2
1–2	1.5
2–3	1.4
3–5	1.1
5–7	0.8
7–17	0.7
2. *Hypercalciuria -* We will identify hypercalciuria using calcium:creatinine ratios defined using age-specific norms and thresholds (Table 3) [64–66]. Measurements will be performed on urine collected in the operating room immediately prior to surgery and on the first postoperative day.

#### Vitamin D axis function

We will evaluate vitamin D axis function through changes in 1,25(OH)_2_D [22] levels from blood collected at specified times following surgery. Based on our previous work, we anticipate a 40 % intra-operative decline in 1,25(OH)_2_D levels [8] and some children will experience a transient decline in 1,25(OH)_2_D levels into the deficient range (<50 pmol/L). Given adequate 25OHD levels and an otherwise properly functioning vitamin D axis, we anticipate restoration (or maintenance) of active hormone levels into the normal range within 12 hours of surgery. Impaired vitamin D axis function will be defined as an inability to maintain active hormone levels in the normal range at any point from blood work collected after the first postoperative day.

#### Organ function and ICU outcome measures

Postoperative cardiovascular and immune function will be measured and compared between the two groups. Cathelicidin levels, an endogenous antimicrobial peptide, will be used as a surrogate measure of innate immune function [40, 41, 67]. Clinically relevant measures of cardiac organ function including echocardiograms (for example, ejection fraction), inotrope requirements (for example, vasopressor need or maximum inotrope score) and fluid resuscitation (positive fluid balance in first 48 hours). Further, we will also evaluate standard PICU clinical outcome measures including time to extubation and PICU and hospital lengths of stay.

#### Phase III study feasibility

We will determine the feasibility of a subsequent multicenter large interventional study through an evaluation of study recruitment rate, proportion of protocol violations, proportion of adequate allocation concealment and blinding, proportion of study drug compliance, and proportion of study drop out.

### Study procedures

**Fig. 1:**
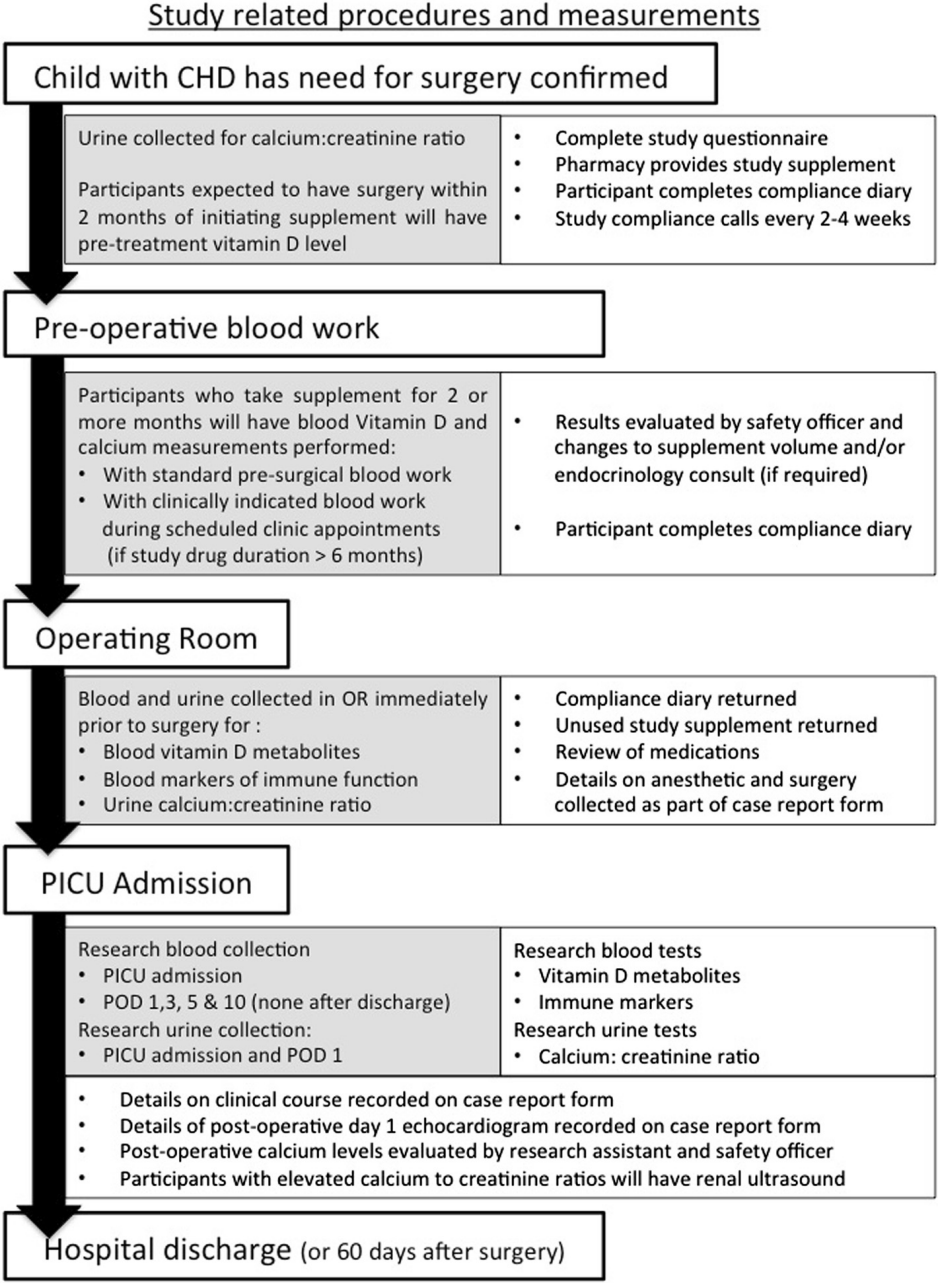
Flow chart of study-related procedures and measurements. Patients and/or caregiver may decline initiation blood work and urine samples and still participate. Postoperatively, urine will only be collected at PICU admission if it was not collected in the operating room. Participants with isolated postoperative hypercalciuria will only have renal ultrasound at the request of nephrology. CHD, congenital heart disease; PICU, Pediatric Intensive Care Unit; POD, postoperative day

A summary of study procedures, biological sample collection and metabolite measurements has been provided as a flow diagram (Fig. 1) and the biochemical measurements on research specimens are summarized in Table 4.

**Table 4:** Biochemical measurements on research specimens

Sample	Timepoint				
	Pre-treatment^a^	During study drug administration^b^	Preoperative^c^	Operating room prior to surgery	Postoperative
Blood	Vitamin D^d^	Vitamin D^d^	Vitamin D^d^	Vitamin D^d^	Vitamin D^d^
		Ionized calcium	Ionized calcium	Ionized calcium	Ionized calcium
				VDBP	VDBP
				PTH	PTH
				Immune function	Immune function
				(for example, cathelicidin)	(for example, cathelicidin)
				Immune activity	Immune activity
				(for example, cytokines)	(for example, cytokines)
Urine	Calcium to	-	-	Calcium to	Calcium to
	Creatinine ratio			Creatinine ratio	Creatinine ratio

^a^neonates, or < 2 months of supplement anticipated

^b^any patient who receives > 6 months of supplement

^c^all outpatients who receive > 2 months of supplement

^d^25OHD will be measured to indicate vitamin D status. The 25OHD level determined at the time of standard preoperative blood work, for safety purposes, will be ordered through the laboratory. The intraoperative and postoperative 25OHD measurements will be determined, using LC-MS/MS technology that adheres to and meets the standards of the Vitamin D External Quality Assessment Scheme

#### Prior to initiation of study drug

**Urine** - After consent is obtained and the study participant is waiting for the pharmacy to prepare the study drug, we will gather a urine sample for determination of calcium:creatinine ratios. Where developmentally appropriate, the participants will be asked to provide urine into a container. Urine bags will be placed on younger children. If the child is unable to provide a urine sample during the time it takes to fill the study drug prescription, the participant will be provided with a container or urine bag and asked to provide an outpatient urine sample.

**Blood** - Neonates and other study participants requiring surgery within 2 months of diagnosis and enrollment will have 0.5 to 1 mL of blood collected prior to (or within 2 days) of starting the study supplement for determination of 25OHD. Where possible, unused or discarded blood will be obtained from the laboratory [68]. If unused blood is not available, research blood will be collected at the time of clinically indicated blood work, or not at all. These patients will not have blood collected for research purposes again until they are taken to the operating room.

* Note - We will request initiation samples but children will still be enrolled if these samples cannot be collected or the families do not want these procedures.

#### During period of study drug administration

All outpatient participants will have blood collected at the time of standard presurgical blood work (2 to 3 weeks prior to surgery) for both 25OHD and ionized calcium. These samples are collected to ensure that patients do not go for surgery with potentially toxic levels of vitamin D. The safety officer will review the ionized calcium level on the patients chart, and follow up if required, as per the safety measures outlined in the sections below. For those participants who will receive the study drug for more than 6 months, we will measure 25OHD and calcium, and they will also have additional blood work to ensure that potentially toxic levels are not maintained for long periods prior to surgery. The timing of this blood work will not be specified and will occur as part of clinically indicated blood work during regularly scheduled clinic appointments

#### Intraoperative biological samples and measurements

**Blood** - All study participants will have 2 mL of blood collected in the operating room following anesthesia and intubation, but prior to skin incision and initiation of cardiopulmonary bypass. Preoperative ionized calcium will be determined. Remaining sample will be aliquoted and stored at -80 °C for determination of 25OHD at the end of the study.

**Urine** - All study participants will have urine collected after insertion of the urinary catheter and the calcium:creatinine ratio will be determined. Study results will not appear on the patient hospital chart, but will be labeled with the study ID number and forwarded to the study investigator and safety officer for review.

#### Postoperative biological samples and other study measurements

**Blood** - All study participants will have 2 mL of blood collected following separation from cardiopulmonary bypass (at admission to PICU). Table 4 shows the biomarkers to be measured. Further, study participants will have 2 mL of blood collected on postoperative days 1, 3, 5 and 10 in the PICU. Samples will be collected from arterial or central venous catheters at the time of clinically indicated blood work. If these catheters have been removed, blood will be collected at the time of clinically indicated venipuncture. If patients are discharged to the ward before the day 10 research sample is collected, a discharge sample will be collected at the time of discharge and no further research blood will be gathered. To limit the volume of research blood collected, neonates will only have 1 mL of blood collected postoperatively on days 3, 5, and 10.

**Urine** - All study participants will have urine collected from the urinary catheter on the first postoperative day. Calcium and creatinine concentrations will be determined.

**Echocardiography** - A comprehensive exam will be performed immediately postoperatively (standard of care) and on the first postoperative day by a trained technician or pediatric cardiologist. Between-group comparisons will evaluate for differences in left-ventricular in (LV) end-diastolic diameter, LV end-systolic diameter, and LV ejection fraction [34].

#### Case report form

All trial information will be stored in a secure electronic database to maintain confidentiality. Data entry methods are in place to promote data quality. In addition, protocol deviations, including discontinuation of study participation, will be recorded. The case report form will be developed using REDCap [69]. Research Electronic Data Capture is a secure web application for building and managing online surveys and databases. The following data will be entered electronically:

1. *Questionnaire -* On the day of surgery the research coordinator will collect the participant dairies and unused study supplement. The patient diaries will contain information on which days the patient was given the study drug, why not, and whether there were difficulties. Information will also be collected on prescribed medications, nutrition, additional supplement use, and symptoms associated with vitamin D intoxication (for example, constipation or abdominal discomfort).
2. *Operative details -* The research assistant will extract detailed operative information, including the following: cardiac lesion type, surgery performed, RACHS score [70], total fluid intake and output, blood product and fluid administration and loss, hypothermia, need for deep hypothermic circulatory arrest (duration), aortic cross clamp times, CPB circuit volumes, CPB circuit constituents, CPB time, occurrence of intraoperative hyper or hypocalcemia, administration of parenteral calcium, need for catecholamines following separation from CPB, and occurrence of intraoperative arrhythmias.
3. *PICU course -* Clinically relevant information on clinical course and organ dysfunction will be collected, including: death, ECMO, PRISM illness severity [71], cardiovascular dysfunction (fluid bolus requirements, inotrope/catecholamine use, arrhythmia), renal dysfunction (urine output, creatinine measurements, need for dialysis), hypocalcemia and calcium administration, duration of mechanical ventilation and duration of PICU stay.

### Statistical analysis

#### Sample size justification

Based on our observational studies and findings from recent dose evaluation studies on healthy children, we estimate that no more than 40 % of the usual care arm will have postoperative 25OHD levels above 50 nmol/L. Based on the 25OHD levels achieved with 1600 IU/day in recent studies on approximate IOM high dose in healthy children, we anticipate that 80 % of the high dose arm will have postoperative levels above 50 nmol/L. Therefore group sample sizes of 28 in both treatment arms will be required to achieve 80 % power to detect an absolute difference between the group proportions of 0.40. The test statistic used is the two-sided Fisher’s exact test and the significance level of the test was targeted at 0.05. Assuming a 10 % drop-out rate, approximately 62 patients (total) will need to be recruited.

#### Comments on power for evaluating vitamin D-related adverse outcomes

**Hypercalcemia** - Our previous observational study (n = 58) identified no cases of preoperative or immediate postoperative hypercalcemia [26]. With a baseline rate in the usual care arm between 0 and 10 %, our sample size would be sufficient to show a statistically significant absolute difference between groups if the rate in the high dose arm exceeded 30 %.

**Hypercalciuria** - Information on baseline rates of hypercalciuria prior to or following cardiac surgery with usual care vitamin D intake is not available. The proposed sample size would be sufficient to demonstrate a 35 % absolute difference in proportions if the baseline pre- or postoperative rates are up to 20 %.

#### Statistical procedures

The analyses will be conducted using SAS software (Copyright SAS Institute Inc., Cary, NC, USA) and a *P* value less than 0.05 will be considered statistically significant.

#### Descriptive statistics

Treatment groups will be described and compared using (i) means with standard deviations or medians with inter-quartile range values for continuous variables or (ii) frequencies with percentages for categorical variables. Statistically significant differences will be determined using Chi-square and Fisher’s exact tests for categorical variables, and t-tests or nonparametric tests (for example, Wilcoxon) for continuous variables, as appropriate.

#### Primary outcome

The primary analytical approach will be to evaluate all randomized patients in an intention to treat analysis. Differences in the primary outcome measure, proportion with 25OHD <50 nmol/L, between the treatment groups will be evaluated using the Fisher’s exact test. Logistic regression analysis will be used if important variables are unevenly distributed between groups. We anticipate minimal missing data because more than 95 % of participants from the recently completed observational study had an immediate postoperative sample [26].

#### Secondary outcomes

Secondary analyses will be evaluated between groups based on data type. Outcome measures that are continuous will be evaluated using the *t*-test, Wilcoxon sign rank test (where appropriate) or through linear regression analysis if important variables are not evenly distributed between groups. Binary secondary outcome measures (for example, hypercalcemia, hypercalciuria) will be compared between the two treatment groups using Fisher’s exact or Chi-square. For the analysis of outcomes measures that represent time to event (for example, restoration of 1,25OH_2_D levels to normal range, time to extubation, and PICU length of stay/discharge), we will apply the log rank test. If randomization does not lead to equal distribution of important variables (for example, weight) the analysis will be expanded to multiple regression modeling (for example, logistic, linear, Cox proportional hazard).

#### Subgroup analysis

The well-known pharmacology of enteral vitamin D dosing shows that up to 2 months of regular daily intake is required to build body stores and achieve steady state blood levels of vitamin D. Consequently, neonates or other infants enrolled into the study who receive surgery within two months of birth or CHD diagnosis will be analyzed separately. Within this subgroup analysis, the primary objective remains reporting of the proportions (in the usual care and high dose groups) that are vitamin D deficient postoperatively. However, given that these participants will receive study drug for a very short period, we anticipate that the proportion with 25OHD levels above 50 nmol/L will remain low in the high dose group. Our program goal at this stage is to identify a dosing regimen that prevents postoperative vitamin D deficiency in 75 % of CHD patients. Given this goal, and an estimated prevalence of 25 % we would need 12 neonates (or children who receive <2 months) to generate a confidence interval that excludes 75 %.

*Feasibility—*Most neonates with CHD who require cardiac surgery within the first few weeks of life have serious cardiac lesions that can limit enteral nutrition and medication delivery. Anticipating that most of these patients will not significantly elevate 25OHD levels with daily enteral intake at IOM high dose, this study will provide important information on the willingness of health care providers to provide enteral study drug. This information will allow us to consider alternative dosing regimens for future studies based on single or divided doses representing one or more months worth of daily dosing (for example, 5 to 10,000 IU/kg). Only those children completing the entire study protocol will be included in analyses.

### Data and safety monitoring

In order to assess possible changes in risk/benefit ratio to study subjects and to obtain independent oversight of study conduct, an external Data and Safety Monitoring Board (DSMB) will be established to oversee the progress of the study. The DSMB will be composed of representatives from statistics, nephrology and endocrinology. External DSMB study reviews will be conducted after half of the participants (n = 30) have completed all study procedures. The DSMB will review and monitor the study procedures and potential risks with a focus primarily on safety. Serious Adverse Events (SAEs) will be reviewed by the DSMB members in order to determine whether additional safety measures should be initiated. There are no predefined criteria for stopping the study, although the DSMB may recommend changing study drug concentration or stopping the study based on SAE or 25OHD data. If there are significant deviations from major study assumptions, the DSMB or study investigators may choose to evaluate 25OHD levels and stop the study early.

The principal investigator and his co investigators will be responsible for maintaining and assessing subject safety in the study, monitoring the presence and severity of adverse events, and monitoring compliance with study drug use. Information on adverse events will be obtained by laboratory findings, including 25OHD, ionized calcium and urine calcium:creatinine levels.

### Safety measures and clinically relevant research findings

A flow diagram depicts the safety measures in place and response to clinically relevant research findings for individual study participants (Additional file 3). A standard operating procedure has been developed for adherence to the following safety measures.

To avoid vitamin D overdose, hypercalcemia and side effects, we have selected a supplement level recently proven to be safe in healthy children and will target the period of high-dose supplementation to 6 months and no more than 12 months. Study participants with elevated blood calcium and/or vitamin D levels will be identified and contacted by the safety officer. For 25OHD, although 500 nmol/L is generally considered the definitive acute toxicity threshold, we have chosen to intervene with 25OHD levels above 200 nmol/L as this value is supraphysiological and exceeds our study goal. The following details the actions that will be taken with abnormal values:

1. For 25OHD above 200 nmol/L with evidence of hypercalcemia (vitamin D toxicity), discontinue study drug immediately, repeat the values (fasting), and refer to endocrinology.
2. For 25OHD above 200 nmol/L without hypercalcemia, the study drug will be reduced by 50 %; (c) For 25OHD above 250 nmol/L, without hypercalcemia: study drug will be discontinued.
3. For hypercalcemia with 25OHD under 200 nmol/L, repeat the bloodwork (fasting) and refer to endocrinology.

Both postoperative hypo- and hypercalcemia will be managed by the clinical team as required. Study participants with persistently elevated blood calcium levels (for more than 2 days, not explained by intravenous calcium administration) will be referred to endocrinology (Additional file 3).

As prolonged exposure to hypercalciuria (>3 months) could theoretically cause nephrocalcinosis, we will have ultrasounds performed prior to hospital discharge on all patients with elevated immediate preoperative urine calcium to creatinine ratios. Any study participant with nephrocalcinosis will be referred to the nephrology service for further assessment.

Participants that have study drug discontinued or decreased will be retained in the study, have perioperative biological samples collected as outlined, and will be included in the analysis using intention to treat methodology.

## Discussion

Research by our group and others has documented not only that 4 out of every 5 CHD patients have inadequate blood levels of vitamin D following surgery, but an association between immediate postoperative hormone levels and clinical course [22, 25, 26]. Altogether, these findings and similar results in adult critical care and CHD surgery populations, suggest that optimization of vitamin D status following CHD repair could lessen inflammation, reduce nosocomial infection and improve cardiac function [11, 13–17, 43–45]. As an inexpensive medication (approximately $15/month) that is generally regarded as safe, vitamin D has the potential to be an ideal intervention for improving outcomes following CHD repair. As protocols and guidelines should be evidenced-based, well-designed clinical trials are essential to adjust clinical practice. This trial aims to determine whether a preoperative proposed dosing strategy will be sufficient to elevate preoperative 25OHD and prevent postoperative vitamin D deficiency. Further clinical study will be required to investigate how normalization of vitamin D levels impacts the clinical course of patients with CHD requiring cardiac surgery.

### Trial status

At the time of writing, 62 % of the target sample size had been enrolled and the anticipated study completion date is January 2016.

## Additional files

Additional file 1:
**SPIRIT 2013 Checklist: Recommended items to address in a clinical trial protocol and related documents*.** Study: Prevention of vitamin D deficiency in children following cardiac surgery: a study protocol for a randomized dose evaluation trial. (DOC 122 kb) Additional file 2:
**Consent and assent forms.** (PDF 645 kb) Additional file 3:
**Flow chart of study-related safety measures.** (JPEG 166 kb)

## Abbreviations used

AI: Adequate Intake
CHD: congenital heart disease
CPB: cardiopulmonary bypass
DSMB: data safety monitoring board
RCT: randomized controlled trial
RDA: recommended dietary allowance
PICU: Pediatric Intensive Care Unit
PTH: parathyroid hormone
SAE: severe adverse event
UL: tolerable upper intake level
VDBP: vitamin D binding protein
1,25(OH)2D: 1,25 dihydroxyvitamin D
25OHD: 25 hydroxyvitamin D.
