# Supplementary material for: Prevention of vitamin D deficiency in children following cardiac surgery: study protocol for a randomized controlled trial
Source: Trials. 2015 Sep 9;16:402. doi: 10.1186/s13063-015-0922-8 (PMC4564959; doi:10.1186/s13063-015-0922-8)
Supplement: Additional file 2: — Consent and assent forms. (PDF 645 kb) [file 13063_2015_922_MOESM2_ESM.pdf]

Patient Information and Consent Form

Prevention of Post-Cardiac Surgery Vitamin D Deficiency in Children with Congenital Heart Disease: A Pilot Dose Evaluation Randomized Controlled Trial.

Study Doctor: Dr. Dayre McNally

Other Doctor's Involved: Dr. Kusum Menon, Dr. Gyaandeo Maharajh, Dr. Margaret Lawson, Dr. Stephanie Redpath, Dr. Pavel Geier, Dr. Jane Lougheed, Dr/ John Smythe

Address: Pediatric Intensive Care Unit, CHEO,
401 Smyth Road, Ottawa, ON K1H 8L1

Phone Number: 613 - 737-7600, ext 3553

Our research group would like to invite you to take part in a research study on vitamin D. You are being invited to participate because your child is less than 18 years of age and is scheduled to have surgery for congenital heart disease (CHD) at the Children's Hospital of Eastern Ontario (CHEO).

Your decision to participate or not in this study will not affect the care your child receives at CHEO. Before agreeing to participate in this study, it is important that you read and understand the following explanation of the proposed study procedures. The following information describes the purpose, procedures, benefits, discomforts, risks and precautions associated with the study. It also describes your right to decline to participate or withdraw from the study at any time. In order to decide whether you wish to participate in this research study, you should understand enough about its risks and benefits to be able to make an informed decision. This is known as the informed consent process. Please ask the study doctor or study staff to explain any words you don't understand before signing this consent form. Make sure all your questions have been answered to your satisfaction before signing this document. If you agree to have your child participate, you will receive a copy of this form to keep for your records.

Background and purpose of study

Vitamin D is a nutrient and hormone best known for its role in maintaining body calcium levels and bone strength. Recently, vitamin D has been reported to also be important for maintaining the health of the heart, lungs and immune systems. Recent research, including work at CHEO, has shown that 4 out of every 5 children with Congenital Heart Disease have low levels of vitamin D following

cardiac surgery. Further, this research demonstrated that children have low levels due to borderline normal levels before surgery and close to a 50% drop due to the life saving cardiac surgical procedures. We are concerned that low levels of vitamin D may contribute to some of the common problems that develop after CHD surgery, including low calcium levels, poor heart function, and inflammation. Our goal is to identify an approach to vitamin D supplementation that safely prevents post-operative vitamin D deficiency.

What is the current standard of treatment for vitamin D?

A minimal daily vitamin D intake has been recommended by the Institute of Medicine and endorsed by Health Canada (400 IU for infants, 600 IU over 1 year). At present, most children with Congenital Heart Disease receive this minimal vitamin D supplement dose prior to surgery. Our research suggests that this approach may not maintain adequate vitamin D following cardiac surgery.

What is the proposed alternative Vitamin D treatment?

The goal of this research study is to evaluate whether a higher daily dose of vitamin D can **safely** prevent vitamin D deficiency following cardiac surgery (1600 IU for infants, 2400 IU over 1 year). **The higher daily dose of vitamin D will be compared to the current standard minimum dose recommended by Health Canada.** The higher dose of vitamin D **we have chosen** is based on results from studies evaluating vitamin D supplementation in healthy children **and recommendations from Health Canada**. These studies showed that children receiving the higher dose were more likely to achieve adequate vitamin D levels and were no more likely to have unwanted side effects (elevated blood and urine levels of calcium). **For children with Congenital Heart Disease** it is unclear whether the higher daily dose of vitamin D will similarly elevate vitamin D levels and avoid unwanted side effects in.

If you agree to participate in this study, your children would be assigned to one of two study groups: one group would receive the low dose of vitamin D (usual care) and the second group would receive the higher dose of vitamin D. This will allow us to compare those subjects who receive the high dose and those who receive usual care. Health Canada and the CHEO Research Ethics Board has given approval to test the safety and effectiveness of the higher dose of vitamin D.

Objectives of study

a. To determine whether the daily intake of a higher dose of vitamin D can decrease the number of children with CHD who have inadequate vitamin D levels following surgery, **as compared to minimum standard vitamin D supplementation recommended by Health Canada.**
b. To determine whether the daily intake of a higher dose of vitamin D supplement significantly increases blood and urine calcium levels.

401 Smyth Road, Ottawa, ON K1H 8L1, Canada
Tel: (613) 737-7600 www.cheo.on.ca
401, chemin Smyth, Ottawa (ON) K1H 8L1, Canada
Tél.: (613) 737-7600 www.cheo.on.ca

c. To determine whether the daily intake of a higher dose of vitamin D supplement improves the functioning of the vitamin D axis.

How many people will take part in this study?

The study doctors will be inviting 62 children with CHD having cardiac surgery at CHEO.

Eligibility to Participate

The doctor in charge of this study, or a member of the study staff, has discussed with you the requirements for your child's participation in the study. Some of the requirements are:

- Your child must be less than 18 years of age
- Your child must have Congenital Heart Disease and require cardiopulmonary bypass and cardiac surgery within 12 months
- Your child will be admitted to the Pediatric Intensive Care Unit (PICU) after surgery.

Your child cannot participate in this study if:

- Your child is known or suspected to have William's syndrome (a rare genetic disorder that can lead to elevated blood calcium levels).
- Your child has a health problem that prevents ingestion of the vitamin D supplementation
- Your child has previously participated in this study.

Study Procedures

Following your decision to participate we will ask you to take either the usual standard daily dose or a high daily dose of vitamin D prior to surgery; when possible the supplement would be introduced a minimum of two months prior to surgery. You will be 'randomized' into one of the study groups described below. Randomization means that you are put into a group by chance. Neither you nor your doctor can choose the group you will be in. You will have an equal (one in two) chance of being placed in any group. The purpose of randomization is to ensure that those receiving the high and low dose of vitamin D supplement are identical in every other respect. That way, we can know for certain that any differences that we observe between the two groups are due to the study medication and nothing else. If your child develops a problem during the course of the study that might be related to vitamin D, the treating physician can request to know the dose of vitamin D your child was assigned.

In this study all participants will receive at least the **Health Canada** recommended minimum daily dose of vitamin D (usual care). **As infant formula contains the minimum daily dose of vitamin D,** some study participants **assigned to the usual care group will** receive a vial that contains a solution without any vitamin D (also called placebo). The placebo is required so that it is not obvious these children are receiving the low dose supplement.

401 Smyth Road, Ottawa, ON K1H 8L1, Canada
Tel: (613) 737-7600 www.cheo.on.ca
401, chemin Smyth, Ottawa (ON) K1H 8L1, Canada
Tél. : (613) 737-7600 www.cheo.on.ca

Making a difference in the lives of children and youth
Faire une différence dans la vie des enfants et des adolescents

Complete a short questionnaire. You will be asked to complete a questionnaire booklet regarding age, current medications, diet and supplements, family background, sun exposure habits and ethnicity. This will take about 5-10 minutes. For outpatient participants this can be completed while you are waiting for the CHEO pharmacy to provide study supplement.

Record supplement intake and permit study staff to contact the caregivers every 2 to 4 weeks. You will be provided with a calendar. We ask that you mark on the calendar every day that your child received the supplement. If the supplement was held, we will ask that you record the reason and duration. You will also be asked to contact study staff if more than 2 days of supplement is held. For those children who are at home prior to surgery we also request permission to have the study staff contact you every 2 to 4 weeks. The purpose of this phone call will be to answer questions related to the study and encourage the daily intake of the supplement.

Permit a small amount of blood to be taken prior to surgery.

1. For neonates, or other children who require surgery within two months of starting the study, we will collect 1 mL or a bit less than a quarter of a teaspoon of blood at the initiation of supplement. To avoid unnecessary discomfort blood will either be collected from arterial or central lines or at the time of venipuncture for clinically indicated blood work.
2. **For children, who have received study supplement for more than 6 months we will measure calcium and vitamin D during scheduled cardiology or cardiovascular clinic appointments if your physician is ordering bloodwork for another purpose.**
3. As part of this study, we will collect blood for measurement of calcium and vitamin D levels at the time of standard pre-surgical blood work **(approximately 3 weeks prior to surgery).** These measurements will be used to make sure children do not have unnecessarily high levels of vitamin D or calcium and allow for adjustment of the supplement dose.

Permit small quantities of blood to be taken in operating room and after surgery. In the operating room, immediately prior to surgery, all patients have lines placed for monitoring and blood collection. Prior to surgery, 2 mL (less than half a teaspoon of blood) will be collected from these lines. Further, after the operation is complete we will take blood at the time of admission to PICU, and on the 1st, 3rd, 5th and 10th post-operative days. If your child is discharged from PICU to the pediatrics ward before day 10, a sample will be collected on the day of discharge and no additional blood will be collected for research purposes. Neonates will only have 1 mL or a bit less than a quarter of a teaspoon of blood collected on the 5th and 10th post-operative days.

Permit small quantities of urine to be taken in operating room and after surgery. In the operating room, immediately prior to surgery, all patients have a tube placed in the bladder for measurement of urine output. This tube remains in place following admission to the Pediatric Intensive Care Unit (PICU). We request permission to collect 5 mL of urine from this tube immediately prior to surgery and once on the day following surgery. This urine will be used to determine whether children who receive the higher dose of vitamin D expel higher amounts of calcium.

401 Smyth Road, Ottawa, ON K1H 8L1, Canada
Tel: (613) 737-7600 www.cheo.on.ca

401, chemin Smyth, Ottawa (ON) K1H 8L1, Canada
Tél. : (613) 737-7600 www.cheo.on.ca

Permit the study doctors to use the collected research blood to determine vitamin D status and measures of vitamin D function. The collected blood will only be used for the express purpose of the research question(s). No other investigations will be allowed without your consent.

Echocardiography (an ultrasound that provides an image of the heart). We request permission to have cardiology evaluate heart function on the day following cardiac surgery through echocardiography. **This often occurs as part of routine care.** This information will be recorded in the research chart.

Permit collection of information from your child' medical chart. Your child's medical chart will be reviewed by the study staff and some information will be recorded (e.g. type of heart disease, duration of surgery, calcium levels, calcium administration, and measures of organ dysfunction).

Additional appointments or changes to care. There are no additional appointments planned as part of the study. With the exception of the study supplement, phone calls and additional blood tests, there will be no other changes to the care provided by the CHEO cardiovascular program. Certain additional (research) tests will be performed to ensure patient safety. If abnormalities are identified with these tests, study participants may be recommended to have additional blood work or to see physician specialists that work in the area of vitamin D (**endocrinologists, nephrologists**).

Study procedures summarized in the attached flow diagram.

Potential Risks

The purpose of this study is to compare the usual daily dose of vitamin D to a higher daily dose. Based on our current knowledge we do not know whether the higher dose of vitamin D will be significantly better than the usual dose in terms of preventing post-operative vitamin D deficiency and side effects. The study would be stopped if we learned that this was not in fact true.

Toxicity with vitamin D supplementation has been described and occurs due to high levels of blood calcium. Vitamin D overdose and hypercalcemia (a high level of calcium in the blood) leads to symptoms such as poor appetite, nausea, vomiting, increased urination, weakness, and nervousness. With very high blood levels, calcium can accumulate in the kidneys leading to impaired kidney function. Vitamin D toxicity has been described in two distinct settings. The first setting involves individuals with rare genetic conditions with abnormal handling of body calcium (1 in 10,000 in the general population). For example, some children with Congenital Heart Disease have a syndrome that makes them more prone to high blood levels of calcium. Patients known or expected to have William's syndrome will be excluded from the study. The second setting involves the intentional or unintentional intake of unnecessarily high vitamin D doses. The table below shows the dose and intake intervals linked to toxicity.

401 Smyth Road, Ottawa, ON K1H 8L1, Canada
Tel: (613) 737-7600 www.cheo.on.ca

401, chemin Smyth, Ottawa (ON) K1H 8L1, Canada
Tél. : (613) 737-7600 www.cheo.on.ca

Dose	Interval of intake	Link to toxicity
>600,000 IU	Single or repeat dosing	Strong
10,000-40,000 IU/day	One to four months	Strong
3,000-10,000 IU/day	Months to years	Weak/unclear

Recent studies on vitamin D supplementation have demonstrated that doses of 1600-2000 IU/day (infants) and 2500-3000 IU/day (above 1 year of age) do not increase risk of high blood calcium concentrations or increase urine calcium levels. However, we do not know whether these doses are safe in children with Congenital Heart Disease who require cardiac surgery.

To compare the effectiveness and safety of the two vitamin D doses we need to collect blood from your child at regular intervals. Blood drawing can cause pain, discomfort, bleeding, bruising or infection at the site of the needle. To avoid these complications research blood will be taken from the venous or arterial lines placed in all of these patients for monitoring. If your child's lines are removed or are not available, we will collect the research blood at a time when they have regular blood work. No patient will receive needle poke solely to collect research blood. Analgesic (numbing or pain blocking) cream can be used to decrease the pain and discomfort of blood tests.

Alternatives to Participating in the Study

Your child does not have to take part in this study to have cardiac surgery. In the absence of other medical conditions, if you choose not to participate your physician will encourage you to take the **Health Canada recommended minimum** daily dose of vitamin D.

Possible Benefits

Your child may or may not benefit from participating in this study. However, our hope is to change clinical practice or to take better care of our children with Congenital Heart Disease in the future. Your participation in this study may eventually provide the study doctors with valuable information about how to safely prevent vitamin D deficiency in children undergoing cardiac surgery for Congenital Heart Disease.

Voluntary Participation

Taking part in this study is entirely voluntary. Your decision to participate or not in this study, will not affect the care you receive at CHEO. We will inform you of any new information that might influence your decision to participate in the research project. You may be asked to sign a revised consent form if this occurs.

Withdrawal from the Study

In some cases, your study participation could be discontinued by the study doctor without your consent, at any time for any of the following reasons.

- The research blood work shows elevated levels of calcium and vitamin D
- The study doctor feels it is in your best interest
- You need additional medication that would interfere with the study
- You do not follow the study staff's instructions

You are free to withdraw your child from the study at any time and there will be no penalty to you or your child. You may also choose to have all of the blood and urine specimens destroyed and information collected as part of the research chart withdrawn. Withdrawing from the study will not affect the care you receive at CHEO.

Confidentiality

Your child's personal information will be kept strictly confidential, except as required or permitted by law. **Studies like this one are regulated by Health Canada and therefore** representatives of Health Canada and the CHEO Research Ethics board will have access to your child's personal information. Your child's medical records may be reviewed by the investigators or delegates, the Research Ethics Board, and regulatory authorities for the purpose of verifying clinical trial procedures and/or data. **CHEO internal monitoring research staff may review your research chart and medical records for quality improvement purposes.** Further, there may be instances where risk to the participant is identified. In this case, information will be shared with appropriate medical personnel in order to initiate care.

You or your child will not be identified in any data publication or presentation of this study. Any personal information about you that leaves the hospital will be coded so that it cannot be identified by name. There is a small risk of unwanted release of information from your research records. Health and research records have been used against patients and their families. For example, in Canada, insurance companies may deny insurance to patient's with a certain illness or those that have genetic risk of disease. Your hospital medical records cannot, however, be released unless required or permitted by law or if you sign a release of information. The researchers of this study will protect your research records so that your name, address and phone number will be kept private. Your child's medical information may be held and processed on a computer in a locked office at CHEO or the CHEO Research Institute.

By signing this consent form, you authorize the record review, information storage and data transfer described above.

If the research uncovers information that might be helpful to your child's current or future health (e.g. high blood calcium or vitamin D levels) the information will be disclosed to you by study

401 Smyth Road, Ottawa, ON K1H 8L1, Canada
Tel: (613) 737-7600 www.cheo.on.ca
401, chemin Smyth, Ottawa (ON) K1H 8L1, Canada
Tél. : (613) 737-7600 www.cheo.on.ca

doctor. This physician will contact you to discuss what these results might mean. This physician may request repeat blood work to confirm the findings. Based on the initial or repeat blood work, this physician may recommend you decrease or discontinue the study supplement and refer you to a physician expert in vitamin D (endocrinologist). The cardiovascular surgery team will be made aware of the need for this additional blood work, change in study protocol, or referral to endocrinology. It is possible that the study doctor may decide that no additional action is necessary.

Study Costs

You will not be charged for any test or research procedure required for this study. You will not be paid to participate in this research study. The vitamin D supplement will be provided to you free of charge as long as you participate in this study. If additional blood work or hospital visits are required you may request compensation and will be provided with a parking voucher.

The study doctor(s) will not receive any financial benefit from your participation in this study.

Compensation

In the event that your child suffers injury as a direct result of participating in this study, normal legal rules on compensation will apply. By signing this consent form, you are in no way waiving your legal rights or releasing the investigators from their legal and professional responsibilities.

Study Results

With the exception of potentially important findings, results from the various study related tests will not be made immediately available to you as they will have no clinical value. At your request, you can receive a copy of the study results at the end of the study.

Questions

If you would like more information, please contact the investigator: Dr. Dayre McNally, Telephone: 737-7600, ext. 3553.

The CHEO Research Ethics Board (REB) has reviewed and approved this research project. The REB is a committee of the hospital that includes individuals from different professional backgrounds. The board reviews all research that takes place at the hospital. Its goal is to ensure the protection of the rights and welfare of people participating in research. The Board's work is not intended to replace a parent or child's judgment about what decisions and choices are best for them. You may contact the Chair of the Research Ethics Board, for information regarding patient's rights in research studies at (613) 737-7600 (3272), although this person cannot provide any health-related information about the study.

Option

The blood samples obtained for this study will be used for the express purpose of the research question(s). We would like your permission to use remaining blood to answer related questions on the importance of nutrition, hormones to heart dysfunction and critical illness.

I agree to have any remaining blood used to answer related research questions ☐ Yes ☐ No

401 Smyth Road, Ottawa, ON K1H 8L1, Canada
Tel: (613) 737-7600 www.cheo.on.ca

401, chemin Smyth, Ottawa (ON) K1H 8L1, Canada
Tél. : (613) 737-7600 www.cheo.on.ca

Patient Information and Consent Form Signature Page

To become a part of this study, you or your legal representative must sign this page. If capable, patients over 14 years of age will be asked to sign this form.

By signing this page, you are confirming the following:

- You have read all of the information in this Patient Information and Consent Form, and you have been offered time to think about it.
- All of your questions have been answered to your satisfaction.
- You voluntarily agree to have your child be a part of this study, to follow the study procedures, and to provide necessary information to the doctor, nurses or other staff members as requested.
- You can freely choose to stop your child from being a part of this study at any time.
- You have received a copy of this Patient Information and Consent Form to keep for yourself.
- If you so wish, you may have a copy of the final study results

____
Name of Subject (printed)

______________________________ _________________________
Signature of Subject (≥ 14 yrs) **Date**

____
Printed Parents Name

_________________________ _________________________
Parental Signature for children <14 years **Date**

____
Printed Name of person conducting consent

_________________________________ _________________________
Signature of person conducting consent **Date**

If the patient is ≤ 14 years old, he/she should be informed about this study at a level he/she can understand. If the patient is able, he/she should give assent to participate in the study and personally sign and date the assent form. This is in addition to the signature of the patient's legal/authorized representative.

401 Smyth Road, Ottawa, ON K1H 8L1, Canada
Tel: (613) 737-7600 www.cheo.on.ca

401, chemin Smyth, Ottawa (ON) K1H 8L1, Canada
Tél. : (613) 737-7600 www.cheo.on.ca

Making a difference in the lives of children and youth

Faire une différence dans la vie des enfants et des adolescents

Study related procedures and measurements

Child with CHD has need for surgery confirmed

Urine collected for calcium:creatinine ratio<br><br>Participants expected to have surgery within 2 months of initiating supplement will have pre-treatment vitamin D level	• Complete study questionnaire<br>• Pharmacy provides study supplement<br>• Participant completes compliance diary<br>• Study compliance calls every 2-4 weeks

Pre-operative blood work

Participants who take supplement for 2 or more months will have blood Vitamin D and calcium measurements performed:<br>• With standard pre-surgical blood work<br>• With clinically indicated blood work during scheduled clinic appointments (if study drug duration > 6 months)	• Results evaluated by safety officer and changes to supplement volume and/or endocrinology consult (if required)<br><br>• Participant completes compliance diary

Operating Room

Blood and urine collected in OR immediately prior to surgery for :<br>• Blood vitamin D metabolites<br>• Blood markers of immune function<br>• Urine calcium:creatinine ratio	• Compliance diary returned<br>• Unused study supplement returned<br>• Review of medications<br>• Details on anesthetic and surgery collected as part of case report form

PICU Admission

Research blood collection<br>• PICU admission<br>• POD 1,3, 5 & 10 (none after discharge)<br>Research urine collection:<br>• PICU admission and POD 1	Research blood tests<br>• Vitamin D metabolites<br>• Immune markers<br>Research urine tests<br>• Calcium: creatinine ratio

- Details on clinical course recorded on case report form
- Details of post-operative day 1 echocardiogram recorded on case report form
- Post-operative calcium levels evaluated by research assistant and safety officer
- Participants with elevated calcium to creatinine ratios will have renal ultrasound

Hospital discharge (or 60 days after surgery)

401 Smyth Road, Ottawa, ON K1H 8L1, Canada
Tel: (613) 737-7600 www.cheo.on.ca

401, chemin Smyth, Ottawa (ON) K1H 8L1, Canada
Tél. : (613) 737-7600 www.cheo.on.ca

Assent Form

Prevention of Post-Cardiac Surgery Vitamin D Deficiency in Children With Congenital Heart Disease: A Pilot Dose Evaluation Randomized Controlled Trial.

Study Doctor: Dr. Dayre McNally

Other Doctor's Involved: Dr. Kusum Menon, Dr. Gyaandeo Maharajh, Dr. Margaret Lawson, Dr. Stephanie Redpath, Dr. Pavel Geier, Dr. Jane Lougheed.

Address: Pediatric Intensive Care Unit, CHEO,
401 Smyth Road, Ottawa, ON K1H 8L1

Phone Number: 613 - 737-7600, ext 3553

Your doctor or nurse will have explained all about this trial to you in a lot more detail than is here, but this information sheet is for you to keep and to help you remember what you have been told. If there are words that you do not understand, please ask your doctor or nurse. You will also be given a separate sheet where some of these words are explained in more detail. You or an adult may contact the research doctor or nurse at 613-737-7600 ext 3553 if you want to discuss any of this further when you get home.

Invitation

You are being invited to be part of a research study. It is up to you if you want to be in this study. No one will make you be part of the study. Even if you agree now to be part of the study, you can change your mind later. No one will be mad at you if you choose not to be part of this study. If you decide not to take part in this study it will not affect the care you receive.

Why Are We Doing This Study?

You are being invited to be a part of this study because you were born with a heart problem that requires surgery within 12 months. We recently learned that the machines used to perform surgery on the heart cause almost all children to have low blood levels of a substance called vitamin D. We believe that having low blood levels of vitamin D following surgery increases risk of infection and places extra stress on the heart and lungs. Our recent study suggests that the usual daily intake of vitamin D recommended for children without heart disease may not protect children who require heart surgery from low blood levels of vitamin D. The goal of this study is to determine whether a higher daily intake of vitamin D can safely prevent low blood levels of vitamin D following surgery.

What Will Happen in This Study?

If you agree to participate, you and your parents will be asked questions about your health, food intake and medications. You and your parents will be given a bottle of vitamin D with instructions to take a certain amount every day prior to surgery. Some children will take a lower amount of vitamin D, while other children will take a higher amount. You and your doctors will not know which group you are in. We will ask you to mark down on a calendar every day you take the study supplement and we will call you every 2 to 4 weeks to answer questions and encourage you to keep taking the study supplement. To compare the children taking low and high amounts of vitamin D we need to do tests on your urine and blood. First, we will ask you to give us some urine prior to starting the study supplement. Second, **we need to make sure your blood levels of vitamin D and calcium are safe. To do this we will do some tests 2 to 3 weeks before surgery (at the same time your surgeon wants blood work to prepare for surgery). For children who take study drug for more than 6 months we will also check the vitamin D and calcium levels during a visit, but only if the heart doctor is doing blood work for other purposes.** On the day of your surgery we will measure your vitamin D levels before and after the surgery. After surgery we will also look for signs that the blood levels of vitamin D were too high or too low. To do this we will need to collect some blood, urine, and take some pictures of your heart (echo). We will also collect information from your medical chart.

Will it Hurt?

- No, we have designed our study so that there will be no additional needles. We will collect the blood required for the tests at the same time as the bloodwork your doctor has ordered.
- We will ask that you give us a sample of urine prior to starting the vitamin D supplement. This will not hurt and we will only ask you to pee into a container.
- As part of the study we will take some images of your heart on the day following surgery. This is called an echo or ultrasound. It will not hurt, but may feel uncomfortable.

How will it Help Me?

We do not know if you will benefit from being in the study. We hope it will help us to find out why some people get this disease and why others don't.

Who Is Doing This Study?

Dr. Dayre McNally and other doctors from CHEO, including your heart doctors, will be doing this study. They will answer any questions you have about the study. You can also call CHEO at (613) 737-7600 Ext 3553 if you are having any problems with your tummy. If you get sick, the study doctors will make sure that you are taken care of.

Who Will Know I Am in the Study?

Only your doctor and people who are involved in the study will know who is in the study. When the study is finished, the doctors will write a report about what was learned. This report will not say your name or that you were in the study. We will make sure that all your medical information is kept private. Your parents and you do not have to tell anyone that you are in the study if you don't want to.

Do I Have to Be in the Study?

You do not have to be in this study if you do not want to. You can have as much time as you want to decide to be part of the study. You can discuss the study with your parents. Your participation in this study is voluntary. No one will be upset with you if you say no. Even if you say yes, and you want to change your mind, it is okay.

Can I stop the Study?

Even if you agree now to be part of the study, you can change your mind later. No one will be upset with you if you choose not to be part of this study.

Will My Information Be Kept Private?

Your identity will remain a secret, unless the law says we have to reveal it, for a very good reason for example, if you are at risk. We will make sure that all your medical information is kept private. Your name will not be used, only your initials. Any personal information that leaves the hospital will be coded so that you are not named.

Who can answer Questions?

The CHEO Research Ethics Board is a group of people who reviews all research that takes place at CHEO. The Board's job is to protect people taking part in research. You may contact the Chair of the Research Ethics Board, if you have any questions at (613) 737-7600 (3272), but this person cannot give you any information about the study.

If you would like more information, please call Dr.Dayre McNally at (613) 737-7600 Ext. 3553.

☐ Check box if verbal assent obtained

	**Yes**	**No**
I have been told about the study in detail	☐	☐
I have had the chance to talk to my Doctor about the study.	☐	☐
I understand that all of the information collected will be kept private.	☐	☐
I understand I will be given a signed copy of this consent form	☐	☐
I understand that I am able to stop being in the study at any time.	☐	☐
I agree to have my private information kept in a computer database which is password protected	☐	☐
I agree to have Dr. McNally or his study staff contact me when further information is needed.	☐	☐

Signature

If I put my name at the end of this form, it means that I agree to be in the study.

____________________	________________________	____________
**Child's Name printed**	**Child's Signature**	**Date**

_____________________	______________________________	____________
**Parental Name printed**	**Parental Signature, children < 14 yr**	**Date**

____

Printed Name of Person Conducting Consent

____ ________________

Signature of Person Conducting Consent **Date**
