# Supplementary figures and images for: Prevention of vitamin D deficiency in children following cardiac surgery: study protocol for a randomized controlled trial
Source: Trials. 2015 Sep 9;16:402. doi: 10.1186/s13063-015-0922-8 (PMC4564959; doi:10.1186/s13063-015-0922-8)

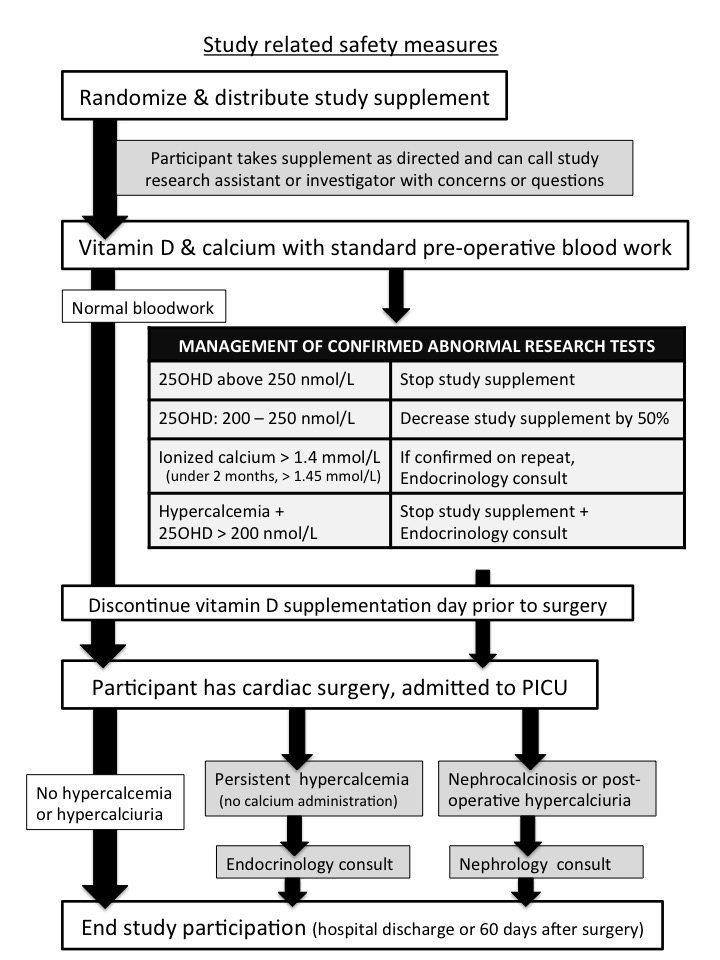

Supplement: Additional file 3: — Flow chart of study-related safety measures. (JPEG 166 kb) [file 13063_2015_922_MOESM3_ESM.jpeg]
